# Gender Difference in Pain Management Among Adult Cancer Patients in Saudi Arabia: A Cross-Sectional Assessment

**DOI:** 10.3389/fpsyg.2021.628223

**Published:** 2021-08-26

**Authors:** Abdulaziz Alodhayani, Khalid M. Almutairi, Jason M. Vinluan, Norah Alsadhan, Turky H. Almigbal, Wadi B. Alonazi, Mohammed Ali Batais

**Affiliations:** ^1^Department of Family and Community Medicine, College of Medicine, King Saud University, Riyadh, Saudi Arabia; ^2^Department of Community Health Science, College of Applied Medical Science, King Saud University, Riyadh, Saudi Arabia; ^3^College of Business Administration, King Saud University, Riyadh, Saudi Arabia

**Keywords:** pain management, gender difference, attitudinal barriers, cancer, Saudi Arabia

## Abstract

**Objective:**

To compare gender differences in pain management among adult cancer patients in Saudi Arabia and to explore the predictors associated with attitudinal barriers of cancer patients to pain management.

**Methods:**

A descriptive cross-sectional study was conducted among 325 cancer patients from tertiary hospitals in Saudi Arabia.

**Result:**

Of the total participants, 67.4% were women (*N* = 219) and 32.6% were men (*N* = 106). The overall mean scores of the attitudinal barriers questionnaire were 49.51 ± 13.73 in men and 54.80 ± 22.53 in women. The analysis shows significant differences in scores in subscales of tolerance (men = 7.48 ± 2.37), (women = 8.41 ± 3.01) (*p* = 0.003) and fear of distraction in the course of treatment (men = 6.55 ± 1.34), and (women = 7.15 ± 2.63) (*p* = 0.008). Female patients reported a more moderate to severe level of pain than men (worst pain in last week of 7.07 ± 1.50, worst pain in last week of 5.84 ± 2.65, respectively). Splitting by gender, the significant predictor for physiology effect domains in male cancer patients includes age, marital status, employment status, monthly income, cancer type, and presence of comorbid disease (*p* < 0.050). Age was a significant predictor of the domains of fatalism, communication, and harmful effects (*p* < 0.050) among female cancer patients.

**Conclusion:**

The present study revealed significant differences between men and women with attitudinal barriers to cancer pain management. Managing pain requires the involvement of all methods in a comprehensive manner, thus unalleviated pain influences the patient’s psychological or cognitive aspect.

## Introduction

Pain is a common and burdensome symptom among cancer patients (van den Beuken-van Everdingen et al., 2007). Approximately, 50% of patients experience moderate to severe pain in the early form to the localized stage of cancer, and around 30% of patients in the advanced form of cancer experience severe cancer-related pain (Miller et al., 2016). Cancer pain is believed to be a significant problem among cancer patients as most of the patients are already terminal upon diagnosis and the fatality of these patients is anticipated to consecutively increase (Al-Shahri, 2009). Cancer pain contributes and leads to poor physical and emotional wellbeing. The goal of the management of cancer-related pain should increase patients’ comfort and improve patients’ functioning (Swarm et al., 2013). Pain management is a critical issue and still a challenge among cancer patients.

Currently, several pain treatment guidelines exist among patients and clinicians that have been directing the course of treatment (Rhee et al., 2012). According to the World Health Organization (WHO), the treatment’s intensity should match the intensification of pain until the optimum degree of individual pain relief is stable by a tolerable level of adverse reaction (Nersesyan and Slavin, 2007). Although there has been significant development in pain management such as cordotomy, rhizotomy, and thalamotomy, it has also been determined that more than fifty percent of cancer patients do not obtain sufficient pain treatment (Koller et al., 2012; Scarborough and Smith, 2018). Hence, it is necessary to improve the quality of cancer pain management and enhance the effectiveness of pain control among cancer patients. Systematically evaluating the methods of cancer pain management should be employed in treating pain among cancer patients (Koller et al., 2012). But despite the different approaches of cancer pain management such as: (1) numerous educational schemes, (2) available pain treatment manuals, (3) therapeutic and non-therapeutic approaches, and (4) formation of integrative pain association, there are still many barriers that have not been adequately defined for cancer pain management. These barriers are possibly associated with the health system, caregivers, and/or patients (Lundorff et al., 2008).

Patient-related barriers to pain management are defined as misguided assessments and misunderstandings that include the feeling of fear of dependence, tolerance, and side effects, reluctance to let physicians try to cure the disease, and belief that cancer pain is an inevitable component in the course of disease (Sun et al., 2008; Jacobsen et al., 2009; Gunnarsdottir et al., 2017). In addition, these might lead to unwillingness or hesitancy to report pain and to use available pain medication such as analgesics (Gunnarsdottir et al., 2017). Moreover, non-adherence with analgesic therapy may result in physicians having to monitor patient symptoms and to see if the medication has relieved their pain (Timmerman et al., 2016).

Prior research involving pain and cancer patients explored the association between patient adherence and gender (Bianco et al., 2011; Birkemeyer et al., 2014; Lauffenburger et al., 2014). The research indicated that gender is an important factor in pain management outcomes (Bianco et al., 2011; Birkemeyer et al., 2014; Lauffenburger et al., 2014). Previous studies reported gender differences in pain in cancer patients (Donovan et al., 2008; Ahmed et al., 2017). For example, chronic pain problems occur mostly among women, and physiologic differences such as hormones are responsible for increased serotonergic activity causing women to be more susceptible to chronic pain disorders (Donovan et al., 2008; Manteuffel et al., 2014). On the contrary, the prescription of analgesics is likely to have a higher dosage among men than women with similar diseases (Manteuffel et al., 2014). Furthermore, the medical outcomes of pain treatments for men and women are significantly different based on the findings of previous studies (Chen et al., 2014). Although previous studies reported gender differences in perceiving pain and in certain medical outcomes, little research has explored the male, and female differences in attitudinal barriers to cancer pain management. Therefore, the purpose of this study was to compare gender differences in pain management among adult cancer patients in Saudi Arabia and to explore the predictors associated with attitudinal barriers of cancer patients to pain management.

## Materials and Methods

### Study Design and Sample Population

The sample population of this descriptive cross-sectional study included 325 patients with a confirmed diagnosis of cancer. Inclusion criteria for the patients were (a) age 18 and older, (b) Saudi nationals, (c) patients with a confirmed diagnosis of cancer, (d) ambulatory and capable patients able to work at least light work, and without restriction, and (e) patients who experienced pain of at least 1 on a range of 0–10 in the past 2 weeks. Ethical approval was obtained from the research ethics committee at King Saud University.

### Data Collection

Data gathering started in January 2019 and was completed in April 2019. Data were collected from three public tertiary hospitals in Saudi Arabia. The selected hospitals were chosen because they are referral hospitals with surgical and oncology specialties established by the Ministry of Health. We distributed 125 questionnaires in each hospital and all eligible participants were recruited using convenience sampling during their visit in the hospital. After attending the medical consultation, all participants were asked to fill in an English-Arabic-translated questionnaire. A trained researcher explained the purpose of the study and their rights in answering the questionnaire. Before the distribution of the questionnaire, approval from the medical directors of the clinics was sought and all attending doctors were asked to set a time to interact, and facilitate the data collection. Written consent was obtained from the respondents before distributing the questionnaire. In addition, all participants were informed before filling out the questionnaire that they could choose to remain anonymous and decline to participate at any time as well as have the option of not completing the questionnaire.

### Instruments and Measures

The questionnaire was designed to measure attitudinal barriers to cancer pain management. The first part of the questionnaire included demographic characteristics and clinical history of the participants. The second part of the questionnaire was the 27-item Barriers Questionnaire II (BQII) which has eight beliefs and four categories:

The eight beliefs are:

• Fear of addiction
• Concerns about tolerance
• Fatalistic beliefs
• Fear from the side effects of medication
• Desire to be a “good patient”
• Fear about interfering and distracting in a physician’s course of treatment
• Concerns about ability to monitor changes in one’s body
• Fear medication may affect the immune system

The four dimensions include:

• Physiologic effect (12 items)
• Fatalism (3 items)
• Communication (6 items)
• Harmful effects (6 items).

The items were measured on a 6-point Likert scale (0 = do not agree to 5 = agree very much. The total score ranged between 0 and 135 and subscale scores were measured by means in which 0 to 1.5 were considered to indicate low levels of concern; 1.5 to 2.5 indicated moderate levels of concern, and means greater than 2.5 indicated high levels of concern (Gunnarsdottir et al., 2005). The Arabic-translated BQII questionnaire had an acceptable Cronbach’s alpha of 0.95. Meanwhile, the BQ-II has been validated in several languages including Japanese, Icelandic, and Norwegian (Gunnarsdottir et al., 2005). The Cronbach’s alpha of the Japanese version of the BQ-II was 0.90 while the Icelandic and Norwegian versions ranged from 0.78 to 0.90 (Gunnarsdottir et al., 2005; Sakakibara et al., 2020).

The third part of the questionnaire was the Brief Pain inventory (BPI) which is a multidimensional pain evaluation tool measuring the presence of pain, intensity, and effect in hindering general activities. The items include questions about feeling of least pain, worst pain, average pain, and pain now during the last 24 h and scored through a visual analog scale (0 no pain to 10 extreme pain) (Bağçivan et al., 2009). The last part of the questionnaire was the Pain Management Index (PMI) which measures the adequacy of analgesic use (Donovan et al., 2008). The Cronbach’s alpha coefficients of the Arabic BPI questionnaire and subscales that have been used varied between 0.91 and 0.99. The PMI was based on the WHO’s analgesic ladder for treating cancer pain and compares patients’ analgesic used and level of reported pain (Donovan et al., 2008). Analgesic usage was determined by four levels (0 = no analgesic; 1 = non-opioid; 2 = “weak” opioid; and 3 = “strong” opioid. The Arabic questionnaire was translated by a professional language translator and pilot tested with 30 patients in the outpatient clinic to ensure the reliability, validity, and its appropriateness and relevance in Saudi Arabia.

### Statistical Analysis

All data were entered and analyzed using the statistical software package SPSS version 23.0 (SPSS Inc., Chicago, IL, United States). Descriptive statistics were used to describe the frequencies of the participants. Independent *t*-tests were employed to examine differences in eight beliefs and domains of attitudinal barriers as well as pain intensity. Then, *t* value, degrees of freedom, and effect size were calculated for each subscale and domains. Additionally, multivariate analysis of variance (MANOVA) was used to assess the differences in attitudinal barriers to cancer pain management for men and women. Linear regression analysis was used with gender as a moderator to determine predictive factors of attitudinal barriers to pain management of cancer patients. A *p* = value was also considered statistically significant when *P* < 0.05.

## Results

Out of 375 questionnaires distributed, 325 participants (86.6%) took part in the study. Table 1 demonstrates the gender differences in demographic and clinical characteristics of the participants. Of the 325 participants, 67.4% were women (*N* = 219) and 32.6% were men (*N* = 106). The analysis shows a significant difference between gender and demographic characteristics such as age, marital status, educational level, and employment status. The number of employed female patients was significantly higher than that of male patients (*P* < 0.01). As can be seen, breast cancer was the most common cancer disease among women (*N* = 112, 51.1%) and colorectal cancer was the most common among men (*N* = 97, 91.5%; *P* < 0.01). In the male group, 37.2% had diabetes mellitus, followed by 8.5% of other types of comorbid disease, and 7.5% had hypertension. Twenty-three percent of women had diabetes mellitus, 8.3% had hypertension, and 5.6% had other types of comorbid disease (*P* < 0.01). The mean Karnofsky Performance Status (KPS) score for men was significantly higher than women (men 67.54 ± 14.80; women 54.01 ± 22.46; and *P* < 0.01).

**TABLE 1:** Patient demographic characteristics.

Characteristic	Men	Women	Total	*p*-value
Total No.	106 (32.6)	219 (67.4)	325 (100)	
**Age, years**				**0.001**
31–40	0	43 (19.6)	43 (13.2)	
41–50	39 (36.8)	59 (26.9)	98 (30.2)	
51–60	48 (45.3)	89 (40.6)	137 (42.2)	
More than 60	19 (17.9)	28 (12.8)	47 (14.5)	
**Marital status**				**0.005**
Single	49 (46.2)	75 (34.2)	124 (38.2)	
Married	57 (53.8)	144 (65.8)	201 (61.8)	
**Educational level**				**0.004**
Non-educated	11 (10.4)	9 (4.1)	20 (6.2)	
Primary	21 (19.8)	22 (10.0)	43 (13.2)	
Secondary	31 (29.2)	78 (35.6)	109 (33.5)	
University – higher	43 (40.6)	110 (50.3)	153 (47)	
**Employment status**				**0.001**
Unemployed	41 (38.7)	85 (38.8)	126 (38.8)	
Employed	65 (31.3)	134 (61.2)	199 (61.2)	
**Income/month**				
>5000SR	54 (51.0)	101 (46.2)	155 (47.7)	0.269
<5000SR	52 (49.0)	118 (53.9)	170 (52.3)	
**Patient status**				
Outpatient	63 (67.9)	175 (99.9)	209 (64.3)	**0.018**
Inpatient	34 (32.1)	44 (20.1)	116 (35.7)	
**Clinical diagnosis**				**0.001**
Breast	0	112 (51.1)	112 (34.5)	
Colorectal	97 (91.5)	28 (12.8)	125 (38.5)	
Other	9 (8.5)	79 (36.1)	88 (27.1)	
**Presence of comorbid disease**				**0.001**
None	49 (46.2)	139 (63.5)	188 (57.8)	
Diabetes mellitus	40 (37.7)	52 (23.7)	92 (28.3)	
Hypertension	8 (7.5)	19 (8.7)	27 (8.3)	
Other	9 (8.5)	9 (4.1)	18 (5.6)	
**Analgesics**				**0.001**
None	34 (32.1)	0	34 (10.5)	
Non-opioids	17 (16)	52 (23.7)	69 (21.2)	
Weak opioids	55 (51.9)	167 (76.3)	222 (68.3)	
Karnofsky performance status score	67.54 (14.80)	54.01 (22.46)	*Mean*−58.43*SD*(21.23)	**0.001**

*Data presented as mean ± SD; P-value significant at P < 0.05.*

The attitudinal barriers to pain management of cancer patients by gender are presented in Table 2. The overall mean scores of the attitudinal barriers’ questionnaire was 49.51 ± 13.73 in men and 54.80 ± 22.53 in women. The analysis shows significant differences in scores in subscale of tolerance (men = 7.48 ± 2.37), (women = 8.41 ± 3.01), and (*p* = 0.003) and fear of distracting in the course of treatment (men = 6.55 ± 1.34), (women = 7.15 ± 2.63), and (*p* = 0.008). The magnitude of the difference of tolerance and fear of distracting in the course of treatment was very large with eta squared = 0.343 and 0.287, respectively. In the domains of attitudinal barriers, it shows that the scores of women (11.52 ± 6.84) in harmful effects was greater than the scores of men (9.25 ± 3.91) (*P* = 0.001) with a very large magnitude of difference (eta squared = 0.407).

**TABLE 2:** Gender differences of mean scores for the BQ-II total scale and subscales.

Subscale	Men	Women	*df*	*t*	*P*-value	Cohen’s d
**Beliefs**						
Fear of addiction	7.50 ± 1.83	7.89 ± 2.67	286	–1.49	0.135	0.170
Tolerance	7.48 ± 2.37	8.41 ± 3.01	257	–3.03	**0.003**	0.343
Fatalistic beliefs	8.10 ± 2.51	7.96 ± 2.90	323	0.41	0.680	0.051
Side effects	15.15 ± 3.18	15.33 ± 4.51	280	–0.43	0.667	0.046
Desire to be a “good patient”	7.04 ± 1.64	6.99 ± 2.77	309	–2.67	0.067	0.021
Fear of distracting in the course of treatment	6.55 ± 1.34	7.15 ± 2.63	322	–2.69	**0.008**	0.287
Concerns about ability to monitor changes in one’s body	6.83 ± 1.62	8.02 ± 3.00	319	–4.62	**0.001**	0.493
Immune system	7.73 ± 2.56	7.93 ± 2.81	323	–0.61	0.537	0.074
**Domains**						
Physiology	21.51 ± 9.41	23.66 ± 12.58	268	–1.72	**0.003**	0.193
Fatalism (reversed scales)	3.36 ± 0.78	3.33 ± 0.93	243	–0.71	**0.033**	0.034
Communication of pain	8.65 ± 2.97	9.60 ± 6.75	320	–1.76	**0.001**	0.182
Harmful effects	9.25 ± 3.91	11.52 ± 6.84	314	−378	**0.001**	0.407
Total_sum	49.51 ± 13.73	54.80 ± 22.53	296	–2.18	**0.001**	0.283

*Data are presented as mean ± SD for continuous variables and frequencies (%) for categorical variables. P-value significant at P < 0.05.*

Table 3 provides a detailed summary of the pain severity and interference of the participants. There was a significant difference between men and women in terms of pain severity (*p* = 0.001). Female patients had a mean score in “worst pain in the last week” of 7.07 ± 1.50, “least pain in last week” of 6.15 ± 1.63, “average pain” had a mean score of 6.35 ± 1.71, and “pain right now” was 6.11 ± 1.63. Meanwhile, male patients had a mean score in “worst pain in the last week” of 5.84 ± 2.65, “least pain in last week” of 4.83 ± 2.55, “average pain” had a mean score of 4.76 ± 5.56, and “pain right now” was 4.62 ± 2.40. Pain interference can be classified as no interference to interferes completely. There was a significant difference between men and women for pain-related functional interference (*p* = 0.001). Male participants reported least more likely functional interference compared to women. For example, in terms of general activity, men had a mean score of 4.60 ± 2.43 while women had a mean score of 6.90 ± 1.60. Other factors had similar trends: Mood (men: 4.66 ± 2.51; women: 6.93 ± 1.57), walking ability (men: 4.66 ± 2.51; women: 6.80 ± 1.53), normal work (men: 4.70 ± 2.42; women: 6.80 ± 2.53), relationship with other people (men: 4.69 ± 2.20; women: 6.72 ± 1.72), sleep (men: 4.61 ± 2.09; women: 6.68 ± 1.78), and enjoyment of life (men: 4.49 ± 2.43; women: 6.93 ± 1.59).

**TABLE 3:** Gender differences between pain severity and pain-related function of the participants.

Subscale	Men	Women	*df*	*t*	*P*-value	Cohen’s d
**Pain severity**						
Pain worst in the last week	5.84 ± 2.65	7.07 ± 1.50	138	–6.94	**0.001**	0.571
Pain least in the last week	4.83 ± 2.55	6.15 ± 1.63	148	–4.82		0.616
Pain average	4.76 ± 2.56	6.35 ± 1.71	157	–4.86		0.725
Pain right now	4.62 ± 2.40	6.11 ± 1.63	158	6.64		0.726
**Pain-related functional interference**						
General activity	4.60 ± 2.43	6.90 ± 1.60	136	–8.56	**0.001**	**1.117**
Mood	4.66 ± 2.51	6.93 ± 1.57	141	–8.14		1.084
Walking ability	4.66 ± 2.51	6.80 ± 1.53	143	–8.07		1.029
Normal work	4.70 ± 2.42	6.80 ± 1.53	163	–7.29		1.037
Relationships with other people	4.69 ± 2.20	6.72 ± 1.72	169	–8.30		1.028
Sleep	4.61 ± 2.09	6.68 ± 1.78	180	–8.76		1.066
Enjoyment of life	4.49 ± 2.43	6.93 ± 1.59	150	–9.42		1.188

*Mild (1–4), moderate (5–6), severe (7–10), (0 = no pain and 10 = pain as bad as you can imagine; 0 = no interference and 10 = interferes completely), 0 = does not interfere, 70 = completely interferes, P-value significant at P < 0.05.*

A MANOVA was performed to investigate the gender difference in attitudinal barriers to pain management of cancer patients (Table 4). Eight beliefs and four domains of pain management were used as dependent variables. The independent variable was gender. There was a statistically significant difference between men and women on the combined dependent variables, *F*(12,312) = 17.46, *p* = 0.001; Wilks’ Lambda = 0.59; partial eta squared = 0.40. When the results for the dependent variables were considered separately, the only difference to reach statistical significance using a Bonferroni adjusted alpha level of 0.027, were tolerance, *F*(1,323) = 11.42, *p* = 0.001; partial eta squared = 0.34; immune system, *F*(1,323) = 9.20, *p* = 0.003; partial eta squared = 0.28; pain intensity index, *F*(1,323) = 48.97, *p* = 0.001; partial eta squared = 0.13; and harmful effects, *F*(1,323) = 10.02, *p* = 0.002; partial eta squared = 0.30.

**TABLE 4:** Multivariate and univariate analyses of variance for gender, and attitudinal barriers to pain management.

				**Beliefs**												
	**Multivariate**				**Tolerance**			**Fatalism**			**Communication**			**Psycho**		
	**Wilks’ lambda**	***F***	***p***	**η2**	***F***	***p***	**η2**	***F***	***p***	**η2**	***F***	***p***	**η2**	***F***	***p***	**η2**

Gender	0.59	17.46	0.001	0.40	11.42	0.001	0.03	0.06	0.799	0.001	4.43	0.036	0.014	3.10	0.079	0.01

		**Beliefs**														
		**Monitor**					**Side effects and distraction**					**Pain intensity**				
		***F***	***p***			**η2**	***F***	***p***			**η2**	***F***	***p***			**η2**

Gender		0.16	0.689			0.001	0.70	0.403			0.02	48.97	0.001			0.13

**Domains**																
	**Physiology**				**Fatalism**				**Communication of pain**				**Harmful effects**			
	***F***		***p***	**η2**	***F***		***p***	**η2**	***F***		***p***	**η2**	***F***		***p***	**η2**

Gender	2.42		0.120	0.01	0.45		0.503	0.01	1.92		0.167	0.01	10.0		0.002	0.03

*P-value significant at P < 0.05.*

Tables 5, 6 presents the predictors associated with attitudinal barriers of cancer to pain management by gender. Splitting by gender, in male cancer patients the significant predictor for physiology effect domains included age, marital status, employment status, monthly income, cancer type, and presence of comorbid disease (*p* < 0.050). Age was a significant predictor of the domains of fatalism, communication, and harmful effects (*p* < 0.050) among female cancer patients. Female and older cancer patients complained of more fatalistic beliefs about cancer-associated pain, and had concerns in reporting to physicians about treating the underlying disease. Furthermore, older, female, and breast cancer patients, and patients with a comorbid disease had more concerns, and fear of drug addiction and the harmful effects of pain medicine to the immune system. Meanwhile, younger male cancer patients had more concerns about physiologic effects than older cancer patients (*p* = 0.009). For both male and female participants, marital status and employment status were significantly associated with all of the subscales of attitudinal barriers (*p* = 0.001). Educational attainment was linearly correlated in the domains of communication among male participants while fatalism was correlated among female participants (*p* = 0.001). The analysis also revealed that the patients’ type of cancer was a significant predictor in all domains of attitudinal barriers (*p* < 0.50). The presence of comorbid disease was found to be significantly correlated with the physiology effect domains and communication domains as well as the harmful effect domain in male cancer patients (*p* < 0.50). In female cancer patients, the presence of comorbid disease was significantly a predictor in the domains of physiologic effect and communication (*p* < 0.50).

**TABLE 5:** Predictors associated with attitudinal barriers to pain management of male cancer patients.

	Physiology		Fatalism		Communication		Harmful effects	
	B (95%CI)	*p*-value	B (95%CI)	*p*-value	B (95%CI)	*p*-Value	B (95%CI)	*p*-Value
Age	−3.48 (−6.0–0.9)	**0.009**	−0.74 (−1.87–0.39)	0.197	−0.03 (−0.12–0.52)	0.426	−0.09 (−0.28–0.96)	0.328
Marital status	7.87 (4.0 to −11.68)	**0.001**	2.71 (1.05–4.38)	**0.002**	−0.01 (−0.13 to −0.12)	**0.030**	0.71 (0.43– 0.99)	**0.001**
Educational level	−9.52 (−12.08–6.96)	0.056	−0.27 (−1.39 to −0.84)	0.630	−0.47 (−0.56–0.38)	**0.001**	−0.57 (−0.76 to −0.38)	0.001
Employment status	4.22 (0.51–5.94)	**0.001**	1.92 (1.17–2.67)	**0.001**	0.24 (0.18–0.30)	**0.001**	0.35 (0.22–0.48)	**0.001**
Monthly income	18.91 (14.04–23.77)	**0.001**	4.74 (2.61–6.87)	**0.001**	0.57 (0.41–0.73)	**0.001**	1.70 (1.34–2.06)	**0.001**
Cancer type	−12.88 (−17.19 to −8.85)	**0.001**	−2.97 (−4.85 to −1.08)	**0.002**	−0.67 (−0.81 to −0.52)	**0.001**	−0.72 (−1.04 to −0.40)	**0.021**
Presence of comorbid disease	2.32 (1.36–3.29)	**0.001**	0.16 (−0.25–0.58)	0.436	0.26 (0.22–0.29)	**0.001**	0.11 (0.04–0.18)	**0.001**

*P-value significant at P < 0.05.*

**TABLE 6:** Predictors associated with attitudinal barriers to pain management of female cancer patients.

	Physiology		Fatalism		Communication		Harmful effects	
	B (95%CI)	*p*-value	B (95%CI)	*p*-value	B (95%CI)	*p*-value	B (95%CI)	*p*-value
Age	1.68 (−0.19–3.56)	0.079	0.77 (0.19–1.35)	**0.009**	0.32 (0.15–0.49)	**0.001**	−0.18 (−0.32 to −0.03)	0.014
Marital status	−8.37 (−11.14 to −5.60)	**0.001**	−2.87 (−3.72 to −2.01)	**0.001**	−0.35 (−0.61 to −0.10)	**0.005**	−1.05 (−1.26 to 0.84)	**0.001**
Educational level	1.68 (−1.23–4.60)	0.257	2.36 (1.46–3.26)	**0.001**	−0.15 (−0.61 to −0.10)	0.262	0.43 (0.21–0.66)	0.001
Employment status	3.86 (2.18–5.54)	**0.001**	0.72 (0.20–1.24)	**0.006**	0.26 (0.11–0.42)	**0.001**	0.48 (0.36–0.61)	**0.001**
Monthly income	−5.70 (−9.29 to −2.10)	**0.002**	−3.11 (−4.21 to −2.01)	**0.001**	−0.46 (−0.79 to −0.13)	**0.006**	−0.74 (−1.02 to −0.47)	**0.001**
Cancer type	1.33 (−0.41 to −3.08)	0.135	0.34 (−0.19 to −0.88)	0.211	0.18 (−0.02 to 0.34)	**0.021**	0.22 (0.09 to −0.35)	**0.011**
Presence of comorbid disease	1.52 (0.68–2.36)	**0.001**	0.11 (−0.14–0.37)	0.365	0.13 (0.06–0.21)	**0.001**	0.03 (−0.02–0.09)	0.281

*95% CI = 95% Confidence interval; P-value significant at P < 0.05.*

## Discussion

Cancer pain is a complex condition with an association of severe and prolonged pain that causes a spiritual crisis, and behavioral, emotional, and physical changes that initiate conflicting effects on patients’ aspect of life (Alqahtani et al., 2015). The incidence of pain during the course of the disease increases from 50% in the early stage to 75% in the advanced stage (Burton et al., 2007). Several studies have revealed gender differences in pain perception. For example, chronic pain was more common among breast cancer patients who went through surgery than lung cancer survivors (Sun et al., 2008; Packiasabapathy and Sadhasivam, 2018). And because of the differences in physiology and psychology, it is essential to understand the gender differences in response to pain management. Health professionals must be well trained with proficiency in pain evaluation and managing procedures, because of their significant part in the development in managing the pain of the patient (Alqahtani et al., 2015).

The results of this study suggest that men and women demonstrate dissimilar degrees of pain management. Female patients were more concerned about the physical effects of cancer and side effects of analgesics than men. Our findings are in line with previous studies in which women displayed greater sensitivity to pain and feeling of unpleasantness than men (Donovan et al., 2008; Manteuffel et al., 2014; Ahmed et al., 2017). Similar results were also found in a study conducted in Taiwan where women were more concerned about the use and side effects of analgesics (Chen et al., 2014). Every patient has a unique response in analgesics therapy, similar to the differences between men and women in response to pain.

On the contrary, male patients show a high level of concern in the fatalism subscale than women. Fatalism is the feeling of hopelessness about pain and its medication (Valeberg et al., 2009). Based on the Hopelessness Theory of Depression by Abramson and colleagues, some individuals exhibit a “depressogenic inferential style” in which one person who encountered a negative life event becomes vulnerable to developing episodes of depression (Helgeson and Tomich, 2005). Having a feeling of hopelessness, fear, and anxiety is a common and normal experience in the course of disease (Yi and Syrjala, 2017). However, if a patient has had this feeling for a long time or continues to have this feeling in day-to-day activities it may lead to clinical depression (Niedzwiedz et al., 2019). Clinical depression is associated with impaired functioning, non-adherence to cancer treatment, and reduced quality of life (Jafari et al., 2018; Niedzwiedz et al., 2019).

With regards to the communication subscale, the findings show that women had moderate concerns compared to men. This finding might be due to the different beliefs and attitudes held by women about cancer. Several factors may contribute to barriers in communication among cancer patients such as age, gender, educational level, and ethnicity (Chaturvedi et al., 2014). The communication section of the pain subscale in this study discussed the apprehension that information of pain disturbs the doctor from handling the latent affliction, and the perception that good patients do not grumble about pain (Bağçivan et al., 2009). Persuasive communication among patients and doctors is the foundation of effective pain management, on the other hand, deficient communication among patients in pain, and their doctors is still a prevalent complication.

Another highlight of this study is the difference of severity in pain between men and women. The findings show that women had a moderate to severe level of pain compared to men. This result was parallel to previous studies in which greater pain severity was found among women than men (Barnabe et al., 2012; Tang et al., 2012; Chow et al., 2017). Although, there are inconsistencies with this result because another study reported that men had more severe pain than women and others found no gender differences (Bartley and Fillingim, 2013). Even though there are contradictions in our findings and previous studies, and barriers are still seen in the present study that continue to hinder optimal pain relief during the course of treatment.

Concerning the factors associated with attitudinal barriers to pain management, it was found that age, gender, marital status, employment status, cancer type, and presence of comorbid disease emerged as significant predictors. This result is in agreement with studies done in China and Jordan which found significant associations between employment status and gender in attitudinal barriers to pain management (Zeng et al., 2020; Al-Atiyyat and Vallerand, 2018). This may be due to the fact that older patients had different fatalistic beliefs regarding pain management and the harmful effects of pain medicine, and drug addiction. Therefore, an educational program is needed that will focus on and be designed for patients’ barriers to pain management particularly in medication adherence and adequacy of analgesic use.

The authors acknowledge some limitations of this study. First, due to the small sample size, the result cannot be generalized to the level of pain among all patients with cancer in Saudi Arabia. Future research should investigate in more detail between-gender differences such as in a qualitative study to determine other important attitudinal barriers. However, these findings may serve as initial information that might be useful for future comparison and for health authorities in assessing the differences between men and women in barriers to pain management of cancer patients. We believe that this study may help in developing or enhancing intervention programs to support and reduce pain for cancer patients. Assessing the statistics data acquired from the study, it is highly recommended that pain treatment exercises be proposed and prescribed for patients with cancer. This might also serve as a criterion for good functioning or comparison if it is possible.

## Conclusion

Managing pain requires the involvement of all methods in a comprehensive manner, thus unalleviated pain influences the patients’ psychological or cognitive aspect. This study revealed that there is a significant difference between gender and attitudinal barriers to pain management of cancer patients, as women tended to have more concerns and feel more intense pain than male patients. Understanding health strategies, accessibility of treatments, and specialized improvement are all fundamentals for optimum management for cancer pain since constant pain is still the main conflict for patients with cancer. Continuous support and additional care is needed particularly among female patients by educating them on pain management that will increase their knowledge and adherence to the treatment as well as eliminating the barriers to pain management. An educational program that will lessen the barriers, particularly any misapprehensions regarding the adverse reaction and consumption of medicines, would be helpful.

### Implication to Clinical Practice

The study findings identify significant differences between men and women in cancer pain management in Saudi Arabia. Hence, this study supports the development of gender-specific interventions to treat cancer-related pain. Several findings can be drawn from the study which could be used within the guidelines when developing specific interventions in managing cancer-related pain; for example, (a) high level of concern of patients about side effects, (b) fear of addiction, and (c) fear that pain medication can affect their immune system. Educational programs about cancer pain medication may help patients to lessen their hesitancy and fear of drug addiction, and the harmful effects of pain medicine. Healthcare providers such as physicians and nurses should also use screening and assessment tools to ensure medication adherence, and review the patient’s beliefs as part of their usual clinical care. A wrong belief or patient misunderstanding about cancer pain medication may decrease analgesic adherence and this influences pain severity, and results in failure to manage cancer pain. These findings may also guide future studies to elucidate the underlying cause for these specific differences in Saudi Arabia.

## Data Availability Statement

The dataset used is locked and stored in the College of Applied Medical Science at King Saud University and can be obtained from the author on reasonable request.

## Ethics Statement

The studies involving human participants were reviewed and approved by Institutional Board at King Saud University. The patients/participants provided their written informed consent to participate in this study.

## Author Contributions

AA and KA: conceptualization, writing–original draft, and data analysis. NA, TA, WA, and MB: facilitate data gathering and writing–original draft. JV: writing original draft and data analysis. All authors contributed to the article and approved the submitted version.

## Conflict of Interest

The authors declare that the research was conducted in the absence of any commercial or financial relationships that could be construed as a potential conflict of interest.

## Publisher’s Note

All claims expressed in this article are solely those of the authors and do not necessarily represent those of their affiliated organizations, or those of the publisher, the editors and the reviewers. Any product that may be evaluated in this article, or claim that may be made by its manufacturer, is not guaranteed or endorsed by the publisher.
